# Emergency department utilization by persons with rheumatoid arthritis: a population-based cohort study

**DOI:** 10.1007/s00296-024-05627-z

**Published:** 2024-06-08

**Authors:** Dani G. Contreras, Patrick McLane, Claire E.H. Barber, Katie Lin, Meghan J. Elliott, Kelsey Chomistek, Shanon McQuitty, Eileen Davidson, Clare Hildebrandt, Steven Katz, Eddy Lang, Brian R. Holroyd, Cheryl Barnabe

**Affiliations:** 1 Department of Community Health Sciences, University of Calgary, Calgary, AB Canada; 2 Emergency Strategic Clinical Network™, Alberta Health Services, Edmonton, AB Canada; 3 Department of Emergency Medicine, University of Alberta, Edmonton, Canada; 4 Departments of Medicine and Community Health Sciences, Cumming School of Medicine, University of Calgary, Calgary, AB Canada; 5 Departments of Emergency Medicine and Clinical Neurosciences, University of Calgary, Calgary, AB Canada; 6 Cumming School of Medicine, University of Calgary, Calgary, AB Canada; 7 Arthritis Research Canada, Vancouver, BC Canada; 8 Alberta Health Services, Calgary, AB Canada; 9 Department of Medicine, University of Alberta, Edmonton, AB Canada; 10 Department of Emergency Medicine, Cumming School of Medicine, University of Calgary, Calgary, AB Canada

**Keywords:** Emergency medicine, Rheumatoid arthritis, Health services research

## Abstract

Some emergency department (ED) visits by persons with rheumatoid arthritis (RA) may be avoidable. This study aims to describe ED use by persons with RA in Alberta, Canada over a 10-year period. Using linked population-based administrative datasets, the annual frequency of ED visits, timing of visits, acuity at presentation assessed (Canadian Triage Acuity Scale (CTAS)), return visits within 72 h, and final disposition were assessed. Most responsible diagnoses assessed by the ED provider were categorized. Between 2008 and 2017, a total of 48,633 persons with RA had 416,964 unique ED visits. There was a 41% relative increase in visits over the study period and within a fiscal year 37% of persons with RA on average attended an ED. Half of the visits were assessed as CTAS 4 ‘Less Urgent’ (31%) and CTAS 5 ‘Non-Urgent’ (19%). No specific diagnosis could be assigned in 36% of visits and RA was listed as the most responsible diagnosis in 2.5% of all visits. Hospital admissions, occurring on average for 14% of ED visits, increased by 15% over the 10 years, and were rare for CTAS 4 (6.4%) and CTAS 5 (1.4%) presentations. Male patients (difference to female 1.2%, 95%CI 0.6, 1.7) and urban patients (difference to rural 8.4%, 95%CI 7.7, 9.2) were more frequently admitted to hospital. Persons with RA have increased ED utilization over time, with a significant volume of less urgent and non-urgent visits. Opportunities for appropriate ambulatory care provision to reduce acute care use should be identified.

## Introduction

Rheumatoid arthritis (RA) is an autoimmune inflammatory joint disease that affects approximately 400,000 people in Canada [1], and is more prevalent among females [1–3]. The majority of healthcare interactions for assessment and monitoring of rheumatoid arthritis (RA) occur within outpatient settings. However, acute care services, such as emergency departments (EDs) and urgent care centers (UCCs), remain important access points for patients with unanticipated concerns related to the disease, its treatment, associated complications, and comorbidities. Over a 13-year study period, persons with RA in Nova Scotia, Canada had 1.55 times higher odds of hospitalization and a higher mean frequency of ED visits annually compared to age and sex-matched general population controls, with this utilization occurring particularly during the first year following the date of index diagnosis [4]. In a cross-sectional study from Nebraska, USA, the leading causes of ED visits by persons with RA included flares (11.7%), cardiovascular-related symptoms (10.4%), injury (6.7%), and fractures (3.6%). Persons with RA were often hospitalized for joint replacements or arthroplasty (6.3%), pneumonia (6.1%), fractures (4.4%), and cardiovascular diseases such as myocardial infarctions (3.8%) [5]. Along with a high frequency of hospitalization, longer hospital stays and costs were driven by the number of comorbid conditions associated with RA [5].

Variations in utilization are reflected in the quality of healthcare that is delivered [6, 7]. Adherence to rheumatology treatment guidelines and providing a high degree of continuity of care reduces hospitalization rates, ED visits, and overall healthcare expenditures [8–10]. In Ontario, Canada, the frequency and costs of ED visits for any cause, musculoskeletal-specific ED visits, and hospital admissions for any cause were lower among persons with RA where their care met four established quality-of-care performance measures (seen by a rheumatologist within 1 year of first RA diagnosis code, annual rheumatology visit, disease-modifying antirheumatic drug dispensation prior to or within 2 weeks of the first rheumatologist visit and annual dispensation) [7]. While EDs and UCCs are essential for unscheduled and/or emergent care access when needed, healthcare costs and utilization can be best optimized by ensuring primary and rheumatology specialist care services are accessible and adhere to quality care measures.

This study was undertaken to gain a better understanding of acute care health service utilization among individuals with RA at a population level. Additionally, the study sought to identify any variations in ED usage based on biological sex and geographic location of residence.

## Methods

Study Design and Time Period: This population-based study used comprehensive administrative health datasets maintained by Alberta Health for the Alberta Health Care Insurance Plan (AHCIP) and Alberta Health Services. These datasets detail each patient interaction with the healthcare system at all sources for the province of Alberta across a population of approximately 4.4 million people, with individual patient file linkage permitted by a recorded Unique Lifetime Identifier. For this study, four datasets were accessed: the Discharge Abstract Database (hospitalizations), Practitioner Claims (outpatient physician visits), the National Ambulatory Care Reporting System (emergency department usage), and the Population Registry (demographic information), beginning in fiscal year 2002/2003 and including up to fiscal year 2017/2018. These datasets do not include race and/or ethnicity identifiers.

Population: A cohort of individuals with a diagnosis of RA was derived by applying a validated case definition [11] to the population-based datasets. The case definition was based on the International Classification of Diseases Ninth Revision (ICD-9) codes recorded in physician claims data, and the Tenth Revision (ICD-10) codes recorded for hospitalization data (ICD-9-CM 714.X and ICD-10-CA M05.X-M06.X respectively). Those included in the cohort had 1 hospitalization or at least 2 practitioner claims in 2 years, but at least 8 weeks apart, for the prespecified RA codes at any timepoint from 2002/2003 to 2017/2018 (97% sensitivity, 77% specificity, 67% PPV, 98% NPV) [11, 12].

Outcome Measures: Data from the National Ambulatory Care Reporting System, reflecting ED and UCC visits, were extracted for outcome analysis. The annual frequency of use of ED and/or UCC services by cases was estimated over a 10-year period (fiscal year 2007/2008 to 2017/2018). Visit characteristics including time of day (daytime 08:00 to 16:00; evening 16:01 to 22:00, night 22:01–07:59) and day of week of the visit, the length of stay in the department, final disposition including return to ED within 72 h were assessed. The severity of presentation to the ED or UCC was assigned according to the Canadian Triage and Acuity Scale (CTAS; 1 Resuscitation, 2 Emergent, 3 Urgent, 4 Less Urgent, and 5 Non-Urgent) [13] and the most responsible diagnosis, recorded at time of discharge from ED, were extracted. Because of the large number of individual diagnoses recorded over a 10 year period (> 500), these were assigned to larger diagnostic groupings by authors who are both rheumatologists and health services researchers (C.B. and C.E.H.B) building upon prior groupings in ED use research [14] (available on request). All analyses were conducted for the case cohort, and stratified by sex (binary, male or female as recorded in health administrative data) and location of residence (binary, urban or rural/remote using 3-digit postal code information).

Data Analysis: Descriptive statistics were applied for frequency data and characteristics of use, such as the mean (SD) and median (interquartile ranges) for continuous data and frequency (percentage) for categorical data. Results were stratified and reported based on sex and location of residence. Student’s t-tests were applied to assess for significant differences by sex and location of residence in continuous data, and Chi-Squared tests were used for categorical data. We concluded significant effects if *p* < 0.05. All analyses were completed using SAS (v9.4; SAS Institute, Cary, North Carolina, USA).

Ethics: The University of Calgary Conjoint Health Research Ethics Board approved the study (ethics ID REB20-0357) and administrative approval from Alberta Health Services was granted in March 2021.

## Results

Cohort Demographics (Table 1): The study population included 48,633 persons with RA during the 10-year study accrual period. For the entire cohort, mean age at the earliest visit was 59.5 (SD 18.3) years, with 67.2% recorded as female, and 77.1% residing at an urban address. There was no evidence for temporal differences in sex and location distributions over the 10-year period (data available by request).

Frequency of ED Visits: From 2007/2008 to 2017/2018, there were a total of 416,964 unique visits by persons with RA. Of persons with RA, 37.4% of persons had a visit each year, and this was consistent over the 10-year study period. The annual mean number of visits per person decreased over the 10-year period, from 1.22 (95% CI 1.17, 1.26) in fiscal year 2008 to 0.92 (95%CI 0.89, 0.95) in fiscal year 2017. There was a 41% relative increase in visits completed by persons with RA relative to the general population over the study period (1.5% in 2008 to 2.1% in 2017).

Visit Characteristics (Table 1): Daytime presentations were more common relative to evening and night presentations with little variation over the 10-year period. There was a similar distribution of presentations by day of the week, with Sunday being the least frequent (12.9% of all visits) and Monday being the most frequent (15.1%). The length of stay in the department increased progressively; in 2008, the median visit duration was 128 min (IQR 58, 303) compared to 179 min (IQR 86, 354) in 2017.

Acuity at Presentation (Table 1): The majority of visits were assessed as CTAS 3 (32.1%) and CTAS 4 (31.3%). The proportion of visits triaged as ‘Urgent’ increased over the 10 year period, from 25.1% of all visits in 2008, to 38.7% in 2017.

**Table 1:** Rheumatoid Arthritis cohort and emergency department/urgent care centre visit characteristics, fiscal years 2007/2008–2017/2018

Characteristic		N (%) unless otherwise indicated
Individual Patients		45,377^±^
Unique Visits		416,964
Age, years		
	Mean (SD)	59.5 (18.3)
	Median (IQR)	61 (49–73)
Sex* (%)		
	Female	30,476 (67.2)
Location of Primary Residence (%)		
	Rural	10,374 (22.9)
	Urban	35,002 (77.1)
Acuity Recorded at Triage (%)^1^		*N* = 415,931 visits
	1 - Resuscitation	2171 (0.5)
	2 - Emergent	47,180 (11.3)
	3 - Urgent	133,672 (32.1)
	4 - Less Urgent	130,166 (31.3)
	5 - Non-Urgent	77,077 (18.5)
	9 - Unknown	25,665 (6.2)
Time of Day of Visit (%)^2^		*N* = 416,656 visits
	08:00–16:00	232,567 (55.8)
	16:01–22:00	116,417 (27.9)
	22:01–07:59	67,672 (16.2)
Day of Week of Visit (%)		*N* = 416,964 visits
	Sunday	53,662 (12.9)
	Monday	62,799 (15.1)
	Tuesday	62,515 (15)
	Wednesday	61,350 (14.7)
	Thursday	60,664 (14.6)
	Friday	61,337 (14.7)
	Saturday	54,637 (13.1)
Length of Stay per Visit, minutes		
	mean, SD	280.8 (520.6)
	median, IQR	151 (70–318)
Disposition After Visit (%)		
	Admitted to Hospital	14.4%
	Of those discharged from an ED visit, proportion with a return visit within 72 h (%)	20.7%

^±^Frequency missing *N* = 3256

*Sex only recorded as male or female in the dataset at time of collection

^1^Frequency missing *N* = 1033

^2^Frequency missing *N* = 308

Visit Diagnoses (Table 2): One-fifth of all visits was for the category “Factors influencing health status and contact with health services”, with 55.9% of these visits being triaged as Non-Urgent. This category is coded for healthcare interactions that do not identify an otherwise classifiable disease, injury or external cause for symptoms, and can reflect limited care or service, or discussions for a problem that is not a disease or injury [15]. Infection accounted for 11.8% of all visits during the study period, of which 32.1% were triaged as CTAS 3, 41.9% as CTAS 4, and 11.9% as CTAS 5. Injuries were responsible for 11.4% of all visits, primarily triaged as CTAS 3 (37.1%) and CTAS 4 (44.4%). Musculoskeletal concerns in general were the most responsible diagnosis for 4.6% of visits, with inflammatory arthritis presentations specifically representing another 2.5% of visits, the majority triaged as CTAS 4 (42.9%) and CTAS 5 (20.0%).

**Table 2:** Most responsible diagnoses frequency and by acuity for emergency department visits, fiscal years 2007/2008–2017/2018

Diagnostic Grouping	Proportion of All Visits	Acuity				
		CTAS 1	CTAS 2	CTAS 3	CTAS 4	CTAS 5
Major Adverse Cardiovascular Events	1.6%	6.7%	38.7%	37.5%	11.0%	2.7%
Dermatologic	1.5%	0%	2.1%	14.6%	40.3%	34.7%
Factors influencing health status and contact with health services	19.4%	0%	0.4%	4.0%	25.3%	55.9%
GI	3.7%	0.3%	13.1%	50.3%	26.1%	6.3%
GU	3.2%	0.2%	9.4%	35.5%	41.2%	8.7%
Infection	11.8%	0.4%	9.2%	32.1%	41.9%	11.9%
Inflammatory Arthritis	2.5%	0%	3.2%	27.9%	42.9%	20.0%
Injury	11.4%	0.3%	7.3%	37.1%	44.4%	7.5%
MSK	4.6%	0%	5.2%	33.4%	42.3%	14.4%
Neurologic	3.4%	0.5%	12.0%	43.2%	30.5%	8.9%
Psychiatric	1.4%	0.3%	16.3%	39.6%	29.3%	9.5%
Renal	1.7%	0.5%	18.2%	51.6%	20.2%	5.9%
Respiratory	3.7%	1.9%	20.3%	40.3%	26.6%	6.7%
Substance Use Disorder	1.3%	0.6%	16.9%	42.6%	28.6%	7.5%
Symptoms, Signs and Abnormal clinical and laboratory findings, not elsewhere classified	16.2%	0.5%	20.8%	47.3%	22.9%	5.4%

Acuity assessed in accordance with the Canadian Triage Acuity Scale(6):1-Resuscitation, 2-Emergent, 3-Urgent, 4-Less Urgent, 5-Non-Urgent

Any grouping with > 1% overall frequency

Disposition: From all visits, the frequency of admission to an inpatient facility from the ED was 14.4% (Table 1), increasing 15% from 2008 to 2017. The most frequent diagnoses resulting in admission (Table 3) were ‘Symptoms, signs and abnormal clinical or lab findings not otherwise specified’ (18.0%), infection (15.2%), injury (9.2%), and respiratory disease (8.2%). Admission rates varied by CTAS score, with 61.2% of CTAS 1, 37.2% of CTAS 2, and 22.3% of CTAS 3 ED presentations being admitted. This is in contrast to CTAS 4 and CTAS 5 presentations, where just 6.4% and 1.4% of visits resulted in admission, respectively. ED or UCC visits for inflammatory arthritis visits resulted in admission just 1.2% of the time. Of persons discharged from an initial ED visit, 20.7% had a minimum of 1 ED return visit within the following 72 h (Table 1). This was primarily driven by infection diagnoses. One third (33.1%) of all ED visits specifically for inflammatory arthritis conditions required a repeat visit within 72 h.

**Table 3:** Frequency of admission, by major diagnostic groupings, fiscal years 2007/2008–2017/2018

Diagnostic Grouping*	Admissions (%) n = 60,127
Symptoms, signs, and abnormal clinical or lab findings, NOS	18.0%
Infection	15.2%
Injury	9.2%
Respiratory Disease	8.2%
Gastrointestinal	7.3%
Major Adverse Cardiovascular Event	6.2%
Renal Disease	4.3%
Neurologic	3.1%
Cerebrovascular Disease	2.7%
Inflammatory Arthritis	1.2%

*Nine most frequent causes, and inflammatory arthritis listed. Full table available upon request

**Fig. 1:**
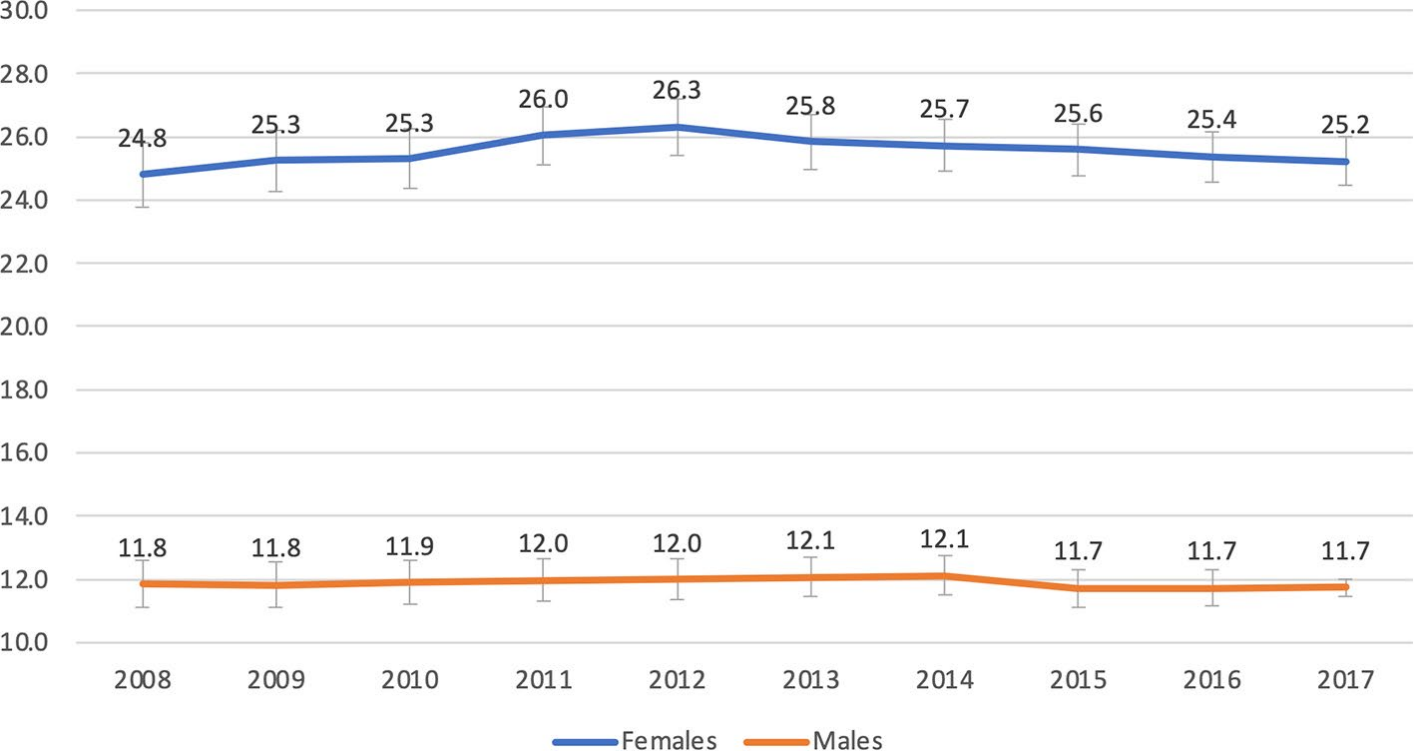
Proportion of RA patients who used an ED/Prevalence of RA, by sex

Sex Differences in ED Use (Table 4): On average 25.5% of females and 11.9% of males with RA had an ED visit within each year (difference 13.5%, 95%CI 13.0, 14.0) (Fig. 1). Female RA patients presented more frequently during the evening shift (28.8% vs. males 26.2%; difference 2.6% 95% CI 2.4, 2.9), while males were more likely to present during the night shift (17.8% vs. females 15.5%; difference 2.3, 95% CI 2.1, 2.6). A higher proportion of females were triaged as CTAS 3 Urgent (32.8% vs. 30.8% males) with male patients more frequently triaged as CTAS 1 Resuscitation (0.7% vs. 0.4% females) or CTAS 2 Emergent (12.4% vs. 10.8% females). The proportion of males admitted to hospital was marginally higher than females (15.0% vs. 14.1%, difference 0.9%, 95%CI 0.7, 1.1), and males were also more likely to return to the ED within 72 h of initial discharge (21.2% vs. females 18.7%; difference 2.5% 95% CI 1.2, 3.8 in fiscal year 2017/2018).

**Table 4:** Sex differences in rheumatoid arthritis cohort and emergency department/urgent care centre visit characteristics, fiscal years 2007/2008–2017/2018

Characteristic		N (%) unless otherwise indicated	
		Female	Male
Individual Patients		30,476	14,900
Unique Visits		280,654	136,309
Age, years			
	Mean (SD)	59.3 (18.5)	60 (17.8)
	Median (IQR)	60 (48–73)	61 (50–73)
Location of Primary Residence (%)			
	Rural	6690 (21.9)	3683 (24.7)
	Urban	23,785 (78.1)	11,217 (75.3)
Acuity Recorded at Triage (%)		*N* = 279,944 visits	*N* = 135,986 visits
	1 - Resuscitation	1268 (0.5)	903 (0.7)
	2 - Emergent	30,358 (10.8)	16,822 (12.4)
	3 - Urgent	91,829 (32.8)	41,842 (30.8)
	4 - Less Urgent	88,763 (31.7)	41,403 (30.4)
	5 - Non-Urgent	50,215 (17.9)	26,862 (19.7)
	9 - Unknown	17,511 (6.3)	8154 (6)
Time of Day of Visit (%)^2^		*N* = 280,421 visits	*N* = 136,234 visits
	08:00–16:00	156,250 (55.7)	76,316 (56)
	16:01–22:00	80,775 (28.8)	35,642 (26.2)
	22:01–07:59	43,396 (15.5)	24,276 (17.8)
Day of Week of Visit (%)		*N* = 280,654 visits	*N* = 136,309 visits
	Sunday	36,057 (12.8)	17,605 (12.9)
	Monday	42,095 (15)	20,704 (15.2)
	Tuesday	42,328 (15.1)	20,187 (14.8)
	Wednesday	41,207 (14.7)	20,143 (14.8)
	Thursday	40,941 (14.6)	19,722 (14.5)
	Friday	41,342 (14.7)	19,995 (14.7)
	Saturday	36,684 (13.1)	17,953 (13.2)
Length of Stay per Visit, minutes			
	mean, SD	281.2 (497.7)	279.8 (565)
	median, IQR	153 (71–320)	147 (67–315)
Disposition After Visit (%)		*N* = 280,654 visits	*N* = 136,309 visits
	Admitted to Hospital	39,661 (14.1)	20,466 (15.0)
	Of those discharged from an ED visit, proportion with a return visit within 72 h (%)*	18.7%	21.2%

*data reported for fiscal year 2017/2018 only

The top five reasons for ED visits were the same for both sexes: (1) Factors influencing health status and contact with health services (females 18.6% vs. males 21.5%; difference 2.9% 95% CI 2.2, 3.7); (2) Symptoms, signs and abnormal clinical and laboratory findings, not elsewhere classified (females 16.4% vs. males 15.8%; difference 0.6% 95%CI -0.1, 1.3); (3) Infections (females 12.2% vs. males 11.1%; difference 1.0% 95%CI 0.4, 1.6); (4) Injury (females 11.3% vs. males 11.5%; difference 0.3% 95%CI -0.4, 0.9); and (5) MSK-related (females 4.7% vs. males 4.2%; difference 0.6% 95%CI 0.2, 0.9). Over the 10-year period, more male patients with an ED visit had “Inflammatory Arthritis” as their most responsible diagnosis (2.6% vs. females 2.4%; difference 0.1% 95%CI 0.1, 0.2).

Geographic Location of Residence Differences in ED Use (Table 5): Urban residents more frequently used an ED annually (26.5% of urban cohort vs. 11.0% of rural cohort members). However, an individual with RA living in a rural area had more than twice the mean number of visits to the ED (14.7 visits, 95% CI 14.2, 15.3) compared to an individual in an urban area (6 visits, 95% CI 5.9, 6.1) over the 10-year study period with variations in time of visit and day of visit. Persons with RA in urban areas stayed significantly longer in emergency departments, with a median of 205 min (IQR 99, 393) than those living in rural areas (median 94 min, IQR 47, 192) (*p* < 0.001).

**Table 5:** Geographical differences in rheumatoid arthritis cohort and emergency department/urgent care centre visit characteristics, fiscal years 2007/2008–2017/2018

Characteristic		N (%) unless otherwise indicated	
		Rural	Urban
Individual Patients		10,374	35,002
Unique visits		169,138	247,817
Age, years			
	Mean (SD)	58.1 (17.4)	60 (18.5)
	Median (IQR)	59 (47–70)	61 (49–74)
Sex (%)		*N* = 10,374	*N* = 35,002
	Female	6690 (64.5)	23,785 (68.0)
	Male	3683 (35.5)	11,217 (32.1)
Acuity Recorded at Triage (%)^1^		*N* = 168,643	*N* = 247,279
	1 - Resuscitation	567 (0.3)	1604 (0.6)
	2 - Emergent	8786 (5.2)	38,393 (15.5)
	3 - Urgent	34,749 (20.6)	98,917 (40)
	4 - Less Urgent	56,262 (33.4)	73,902 (29.9)
	5 - Non-Urgent	50,892 (30.2)	26,185 (10.6)
	9 - Unknown	17,387 (10.3)	8278 (3.3)
Time of Day of Visit (%)^2^		*N* = 169,127	*N* = 247,520
	08:00–16:00	101,493 (60)	131,070 (53)
	16:01–22:00	45,181 (26.7)	71,233 (28.8)
	22:01–07:59	22,453 (12.3)	45,217 (18.3)
Day of Week of Visit (%)		*N* = 169,138	*N* = 247,817
	Sunday	19,831 (11.7)	33,829 (13.6)
	Monday	25,604 (15.1)	37,194 (15)
	Tuesday	26,182 (15.5)	36,332 (14.7)
	Wednesday	25,837 (15.3)	35,512 (14.3)
	Thursday	25,255 (14.9)	35,405 (14.3)
	Friday	25,615 (15.1)	35,722 (14.4)
	Saturday	20,814 (12.3)	33,823 (13.6)
Length of Stay per Visit, minutes			
	mean, SD	184.5 (556.3)	344.4 (485.1)
	median, IQR	94 (47–192)	205 (99–393)
Disposition After Visit (%)		*N* = 169,138	*N* = 247,817
	Admitted to Hospital	15,886 (9.4)	44,235 (17.8)
	Of those discharged from an ED visit, proportion with a return visit within 72 h (%)*	26.1%	17.1%

*data reported for fiscal year 2017/2018 only

A higher proportion of urban individuals were assessed a high triage score (CTAS 1–3) compared to those from rural areas (56.0% vs. rural 26.2%, difference 30.0% 95% CI 29.7, 30.3). Residents from urban areas with RA were significantly more likely to be admitted to hospital than rural individuals (17.8% vs. rural 9.4%, difference 8.5%, 95%CI 7.9, 9.0), while rural residents with an ED visit were more frequently representing within 72 h of initial discharge (26.1% vs. urban 17.1%; difference 8.9%, 95%CI 7.5, 10.3 in fiscal year 2017/2018).

While leading diagnoses for ED visits were similar for rural and urban residents with RA, we found that more rural residents had “Factors influencing health status and contact with health services” coded as their most responsible diagnosis compared to urban residents (29.6% vs. urban 12.7%; difference 16.9% 95%CI 16.6, 17.1). Symptoms, signs and abnormal clinical and laboratory findings was a more common diagnosis in urban patients (rural 11.8% vs. 19.2% urban; difference 7.4% 95% CI 7.2, 7.6), as well as infection (rural 11.6% vs. urban 12.0%; difference 0.31% 95%CI 0.1, 0.5), injury (rural 9.0% vs. urban 13.0%; difference 4.1% 95%CI 3.9, 4.3) and MSK-related events (rural 4.3% vs. urban 4.7%; difference 0.4% 95%CI 0.2, 0.5). Additionally, inflammatory arthritis-related presentations were much higher among rural persons in 2008 (3.5% vs. urban 2.3% difference 1.2% 95%CI 0.9, 1.6) but has decreased over time to 2.3% in fiscal year 2017/2018 (vs. urban 2.4%; difference 0.1% 95%CI -0.2, 0.3).

## Discussion

Interpretation of Findings: This paper describes patterns of ED utilization among persons with RA in Alberta, Canada, including differences by sex and location of residence for utilization. In 2017, individuals with RA accounted for over 48,000 (2.1%) of all ED visits, a notable figure given that the crude prevalence of RA in Alberta is only approximately 1.1% [16, 17].

Over the 10-year study period, nearly half of all visits continue to reflect acute care access for less and non-urgent acuity conditions. These visits contribute to ED patient volumes and potentially overcrowding, rarely result in the need for hospitalization, and add to otherwise preventable costs and burden to the healthcare system. These are highlighted as concerns in the ability to provide safe and timely care [18]. Health service models, developed in collaboration between primary care, rheumatology care and allied health practitioners, are needed to provide reasonable options for access to care for these priority concerns. For example, infections do require urgent attention in persons living with RA, who are likely to be on immunosuppressive therapies and are at risk for rapid deterioration. Access pathways to be assessed for antimicrobial or antiviral treatments outside of EDs, such as through pharmacy providers, could promote efficiency while also providing a method to avoid additional exposures in ED waiting rooms.

Solutions to reduce avoidable acute care use will need to meet the needs of persons based on their membership in biological sex and gender populations, and their location of residence. Males were found to present to EDs at higher acuity levels, required admission more frequently as well as having an increased rate of repeated ED visits. The predominance of evening visits for females likely reflects overlap with societal gender roles of childcare and engagement in the workforce, and speaks to the need for adaptable models outside of traditional ambulatory care physician hours. Rural persons living with RA appear to use the ED for lower acuity concerns, while also having a higher frequency of visit rates despite a lower proportion of the population accessing acute care. This observation could reflect the lack of structured services for particular needs, such as outpatient antibiotic therapy, and the ED becoming a service location for daily treatment. Models selected will need to heed the shortage of rural primary care providers, and utilize technology that connects patients with their specialty care provider urgently to avoid long distance travel requirements.

Comparison to Previous Studies: This study complements other research on ED utilization in universal health care access settings in Canada. In Nova Scotia, the frequency of ED visits by persons with RA was highest during the first year following diagnosis, and decreased over time but remained consistently higher than in the general population [4]. Our results, in a prevalent cohort, also demonstrated a decrease in the average number of visits per individual over time. Given the higher prevalence of RA among females, it is noteworthy that our data estimate a higher frequency of ED visits among females with RA compared to males. This sex-based discrepancy in ED utilization aligns with broader research demonstrating that females tend to have higher ED visit rates and health-seeking behaviours than males [19–22].

Our findings are also in keeping with the literature on comorbidities in persons with RA. It is well established that persons with RA have higher risk of comorbid conditions such as cardiovascular diseases [23, 24], depression [25, 26], osteoporosis [27], and increased frequency of infections [28, 29]. These conditions are known to have acute exacerbations meriting an ED visit and possibly hospital admission. For visits triaged as “Urgent”, major cardiovascular events were the predominant diagnosis. However, we observed a large proportion of visits triaged as ‘Less Urgent’ and ‘Not Urgent’ were coded for circumstances reflecting that a diagnosis for signs and symptoms could not be ascertained, or other factors influencing health status were responsible for the visit. Given the ambiguity of those codes, additional research is required to determine what these visits are for or driven by, and how innovations in models of care and quality care initiatives could be implemented to better align patient needs with the clinical services delivered. This could include addressing physician shortages [30], incentivizing quality care delivery [31], developing care pathways that are patient-centered, and exploring collaborative care models involving interdisciplinary rheumatology care models to ensure broader access to healthcare services [32].

Strengths and Limitations: The strengths of our study include the use of a large population-based dataset with 10 years of available data in a province with universal health care coverage for ED visits with analysis of all interactions with the healthcare system. Utilizing an ED-specific database added the ability to not only estimate the frequency of and most responsible diagnosis for the visit, but also visit characteristics such as time of day and day of week services were accessed, and the acuity at presentation, to inform the structure of potential interventions aimed at reducing unnecessary acute care utilization. Additionally, the use of validated case definitions with optimal specificity reduced potential misclassification of cases. These factors enhance the generalizability of our findings to those with similar health care settings. We also provide stratified results to examine the effects of sex and location of residence on ED use. There are some limitations to our study design. We were not able to access general population control data to compare to the RA cohort. Factors informing disease outcomes, such as disease duration, disease activity, lab and diagnostic imaging data, and medications, and comorbidities that are likely influencing ED use and length of stay in the ED were not available for linkage to cohort data. These factors could additionally explain observations such as reasons for the decline in annual mean number of visits per individual living with RA. We also could not access information about adherence to performance measures in clinical practice and whether patients had access to a family physician or rheumatologist. Our provincial administrative datasets only have information on biological sex, and they do not encompass diverse gender- and ethnicity-related factors influencing ED utilization among individuals with RA. Other characteristics predisposing patients to inequities in care, such as socioeconomic status, could not be studied within the available data. We are currently conducting research to investigate decision making to attend an ED and attempts to access care prior to an ED visit to contextualize our findings.

## Conclusion

The examination of frequency and characteristics of ED use by persons with RA may be used to determine initial targets for improvements to health service models of care. These enhancements must reflect specific needs of patient populations including females and rural residents, who had the highest rates of ED use in our study.

## Declarations

All authors agree to be accountable for all aspects of the work in ensuring that questions related to the accuracy or integrity of any part of the work are appropriately investigated and resolved.
